# Musculoskeletal imaging authority, levels of training, attitude, competence, and utilisation among clinical physiotherapists in Nigeria: a cross-sectional survey

**DOI:** 10.1186/s12909-022-03769-x

**Published:** 2022-10-04

**Authors:** Ogochukwu Kelechi Onyeso, Joseph O. Umunnah, Joseph C. Eze, Ayodele Teslim Onigbinde, Canice Chukwudi Anyachukwu, Charles Ikechukwu Ezema, Ifeoma Uchenna Onwuakagba, Ukachukwu Okoroafor Abaraogu, Agba Peter Awhen, Ernest Emezie Anikwe, Odunayo Theresa Akinola, Michael Ebe Kalu

**Affiliations:** 1 Department of Medical Rehabilitation, Faculty of Health Sciences and Technology, College of Health Sciences, Nnamdi Azikiwe University, Awka, Anambra Nigeria; 2 Population Studies in Health, Faculty of Health Sciences, University of Lethbridge, Lethbridge, Alberta Canada; 3 Department of Medical Rehabilitation, Faculty of Health Sciences and Technology, College of Medicine, University of Nigeria, Nsukka, Enugu, Nigeria; 4 Department of Radiography and Radiological Sciences, Faculty of Health Sciences and Technology, College of Health Sciences, Nnamdi Azikiwe University, Awka, Anambra Nigeria; 5 Department of Medical Rehabilitation, Faculty of Basic Medical Sciences, Obafemi Awolowo University, Ile-Ife, Osun Nigeria; 6 Department of Physiotherapy, Faculty of Allied Medical Sciences, University of Calabar, Calabar, Cross River Nigeria; 7 Department of Physiotherapy, University College Hospital, Ibadan, Oyo Nigeria; 8 Department of Physiotherapy, Faculty of Basic Medical Sciences, College of Health Sciences, Bowen University, Iwo, Osun Nigeria; 9 School of Rehabilitation Science, McMaster University, Hamilton, Ontario Canada

**Keywords:** Clinical competence, Curriculum, Diagnostic imaging, Physical therapists, Referral

## Abstract

**Background:**

Direct-access physiotherapy practice has led to a global review of the use of differential-diagnostic modalities such as musculoskeletal imaging (MI) in physiotherapy.

**Objective:**

To explore the MI authority, levels of training, attitude, utilisation, and competence among clinical physiotherapists in Nigeria.

**Methods:**

This national cross-sectional study analysed a voluntary response sample of 400 Nigerian physiotherapists that completed the online version of the Physiotherapist’s Musculoskeletal Imaging Profiling Questionnaire (PMIPQ), using descriptive statistics, Spearman’s correlation, Mann-Whitney U, Kruskal-Wallis, and Friedman’s ANOVA tests.

**Results:**

Of the 400 participants, 93.2% believed that physiotherapists should use MI in clinical practice. However, only 79.8% reported having MI authority in their practice settings. The participants’ median (interquartile range) levels of training =10 (24) and competence =16 (24) were moderate. Nonetheless, levels of training (χ2 [15] = 1285.899, *p* = 0.001), and competence (χ2 [15] = 1310.769, *p* < 0.001) differed across MI procedures. The level of training and competence in x-ray referral and utilisation was significantly higher than magnetic resonance imaging, computed tomography scan, ultrasonography, scintigraphy, and dual-energy x-ray absorptiometry, in that order (*p* < 0.05). There was a significant positive correlation between the levels of training and competence (rho =0.61, *p* < 0.001). The participants had a positive attitude =32 (32) and occasionally used MI in clinical practice =21 (28).

**Conclusion:**

Majority of the respondents believed they had MI authority although there was no explicit affirmation of MI authority in the Nigerian Physiotherapy Practice Act. Participants had a positive attitude towards MI. However, levels of MI training, competence, and utilisation were moderate. Our findings have legislative and curriculum implications.

## Background

Historically, physiotherapists practised under primary care physicians’ directives [1]. Over time, physiotherapy has evolved into a profession with esoteric knowledge and core clinical competencies [2]. Therefore, World Physiotherapy (WCPT) – the sole international voice for physiotherapy, advocates for an autonomous physiotherapy practice model that involves direct access, the authority to request diagnostic tests, and refer patients to other medical specialities [3, 4]. Musculoskeletal imaging (MI) authority also known as MI request- or referral-right is the legitimate privilege of referring eligible patients for appropriate musculoskeletal diagnostic imaging [3–6]. Clinicians who have MI authority are expected to understand the consequences of using this privilege vis-à-vis the economic costs and the danger of undue exposure of patients to radiation [7].

Accordingly, direct-access physiotherapists are using musculoskeletal imaging to aid clinical decision-making in counties such as Australia, Canada, the United Kingdom (UK), the Netherlands, Norway, and the United States of America (USA) [4–6]. Physiotherapists’ MI authority varies across countries and practice settings [4, 8]. A preliminary survey of physiotherapy MI authorities across the WCPT member nations [6] did not include Nigeria, the authors reported that they were unable to access reliable information from the omitted countries. Knowing the status of physiotherapists’ MI training and authority in Nigeria is an important baseline ahead of the implementation of the newly approved Doctor of Physiotherapy (DPT) curriculum by the National Universities Commission, and the concomitant pursuit of the Practice Act amendment [9]. The DPT curriculum provided a standalone MI course with broader content [9]. Moreover, Nigeria (≈200 million people) is the most populated country in Africa [10], with a type of physiotherapy educational programme and Practice Act typical of most countries on the continent [11]. In Nigeria, physiotherapists working in tertiary hospitals have unrestricted access to patients’ medical imaging reports but rely on primary care physicians for MI referrals [12]. The Nigerian Physiotherapy Practice Act does not include MI affirmative or restrictive language [13].

**Fig. 1:**
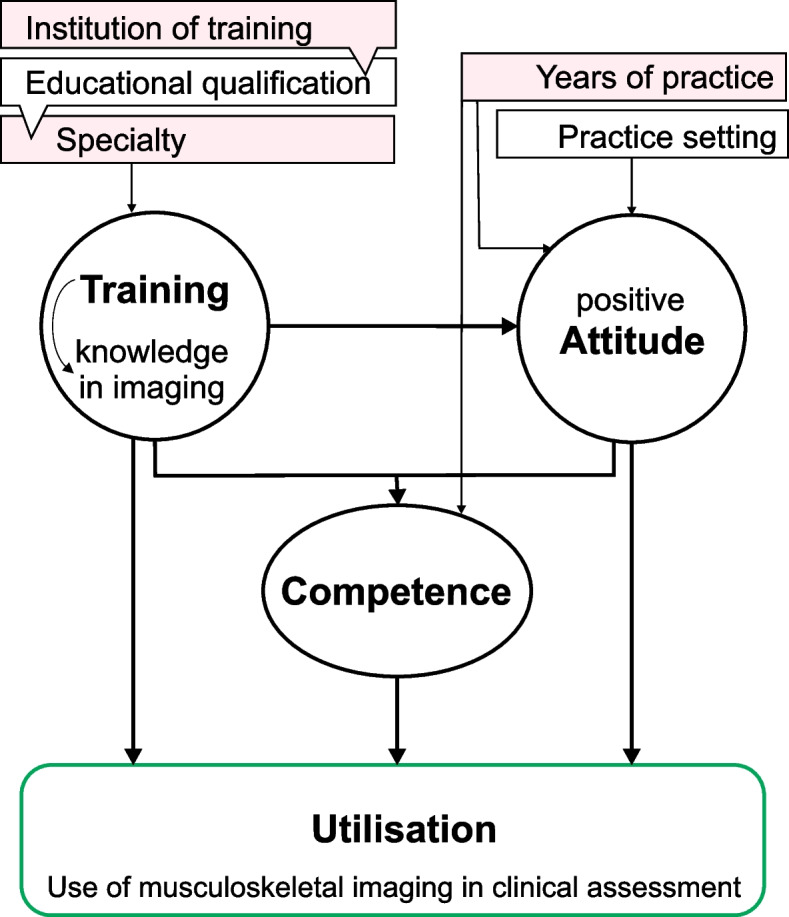
The theoretical framework: Relationship between training, attitude, competence, and utilisation of MI

The literature showed that MI curriculum content, practitioners’ level of education, and speciality impact their overall MI training [14–16]. Moreover, high-quality training, practice setting, and clinical experiences can stimulate a positive attitude, improve competence, and enhance the utilisation of MI skills [9, 15, 17]. We hypothesised that there would be a correlation among levels of training, attitude, competence, and the utilisation of MI in clinical practice (Fig. 1). Our study objectives were to (a) describe the self-reported MI authority, levels of training, attitude, utilisation, and competence in MI among physiotherapists practising in Nigeria, (b) determine the correlation among the levels of training, attitude, utilisation, and competence in MI, and (c) determine the differences in the levels of training, attitude, utilisation, and competence in MI across practice settings, specialities, participants’ years in practice, and educational qualifications.

## Methods

### Study design

This study was a cross-sectional survey of physiotherapists in Nigeria using a standardised online questionnaire [12]. The study protocol was approved by the Health Research and Ethics Committee of the Faculty of Health Sciences and Technology, Nnamdi Azikiwe University, Awka, Anambra, Nigeria (Reference number: ERC/FHST/NAU/2018/198). Informed consent was obtained from all participants. The consent form clearly stated the study objectives, participants’ right to withdraw from the study, data privacy and confidentiality. The study adhered strictly to the approved protocol.

### Study population

The study population comprised all clinical physiotherapists in Nigeria. All physiotherapists in Nigeria are registered with the Medical Rehabilitation Therapists Board (MRTB) – the federal regulatory body for physiotherapy training and practice in Nigeria [13]. At our request, the MRTB sorted the electronic register of all physiotherapists in Nigeria and identified the email addresses of the 2308 physiotherapists who met the inclusion criteria for this study. The inclusion criteria were being a Nigerian-trained physiotherapist who had completed the mandatory post-graduation one-year clinical internship programme and at least one-year post-internship clinical experience, currently licensed and practising in Nigeria [18].

### Sample size calculation

The sample size was calculated from a finite population of 2308 eligible physiotherapists, using the Yamane formula, *n* = N/(1 + N [e]^2^); where the level of significance (e) = 0.05, and sample population (N) = 2308. The minimum required sample size (n) = 364 [2, 18, 19]. However, in anticipation of some incomplete questionnaires, we accepted 436 questionnaires before closing the weblink. After removing 36 surveys with incomplete demographic data, the final analysis was conducted with 400 samples.

### Sampling technique and bias

To minimize sampling bias, all registered physiotherapists who met the eligibility criteria were sent an invitation email. Therefore, each member of the study population had an equal opportunity to participate. Three reminders were sent, and the survey was hosted for an ample period of 30 days [20–22]. However, the actual response to the survey was voluntary, which can lead to the risk of nonresponse bias [23], if an ample study duration and the required sample size were not reached [21].

### Instrument for data collection

The Physiotherapist Musculoskeletal Imaging Profiling Questionnaire (PMIPQ) was used for data collection. The PMIPQ was developed, validated, and pilot tested in Nigeria [12]. The instrument has good psychometric properties; the test-retest reliability score for the domains ranged from 0.72 to 0.98 [12]. PMIPQ has six parts: Part A obtained the demographic information, Part B contained 25 questions regarding the nature of MI training, and Part C, six questions on the level of training on referral and application of results from radiography (X-ray), magnetic resonance imaging (MRI), computed tomography (CT) scan, ultrasound scan, bone scan, and dual-energy X-ray absorptiometry (DEXA) procedures. Part D comprised eight questions on respondent’s attitudes towards the use of MI in clinical practice, Part E, seven questions on respondent’s level of utilisation of MI, and Part F, six questions on the level of MI competence (ability to make MI referral and apply the result appropriately) in musculoskeletal assessment. The participants’ aggregate scores in each domain were interpreted as follows: training score equal to or less than 6 is poor, 7 to 12 is fair, 13 to 18 is good, 19 to 24 is very good, and 25 to 30 is excellent. Attitude score of 8 to 16 is negative, 17 to 24 is neutral, and 25 to 40 is positive. A competence score equal to or less than 6 was regarded as very incompetent, 7 to 12 is incompetent, 13 to 18 is moderately competent, 19 to 24 is competent, and 25 to 30 is very competent. Utilisation score equal to or less than 7 indicates never, 8 to 14 rarely, 15 to 21 sometimes, 22 to 28 most time, and 29 to 35 indicated that the participant always used MI in clinical practice. This paper reported the results from Parts A, C to F, while Part B had been published as an independent paper [9].

### Procedures for data collection

The authors sent a survey invitation email to all the eligible participants in the MRTB database. The email contained the study objectives, participant information sheet, and the weblink to the questionnaire. On accessing the weblink, participants first went through an informed consent page. A participant had to click on the “endorsement” button to proceed to the survey and can choose to exit the questionnaire at any time. Therefore, the return of the completed survey constituted consent to participate in the study [19]. The authors sent three successive reminders to the initial email recipients after 2 days, 4 days, and 7 days. We embedded programming syntaxes in the software to analyse the demographic variables and discard entries from ineligible respondents or multiple entries from eligible respondents. The questionnaire was hosted online for 30 days, between March and April 2019.

### Data analysis

The survey database was downloaded and analysed using SPSS version 26 software (SPSS, Chicago, IL, USA). The descriptive statistics were computed using frequency (percentage) and median (range). Participants’ overall scores in each part (C to F) were treated as continuous variables [24]. Shapiro-Wilk test showed that the variables were not normally distributed, therefore, we used non-parametric statistics for inferential analyses.

Specifically, Spearman’s coefficient (*rho*) was used to test the correlation between the years in practice, levels of training, attitude, competence, and utilisation of MI. Mann-Whitney *U* (reported as *Z*-statistic) and Kruskal-Wallis (*H*) tests were performed to determine the differences in the levels of training, attitude, competence, and utilisation of MI across the demographics. The Dunn-Bonferroni (*Z*) post hoc test was applied when a significant *H*-statistic was obtained. Finally, Friedman’s Analysis of Variance (ANOVA) was employed to test for differences in the levels of training and competence across the MI procedures. The alpha level was set at *p* ≤ 0.05.

## Results

**Fig. 2:**
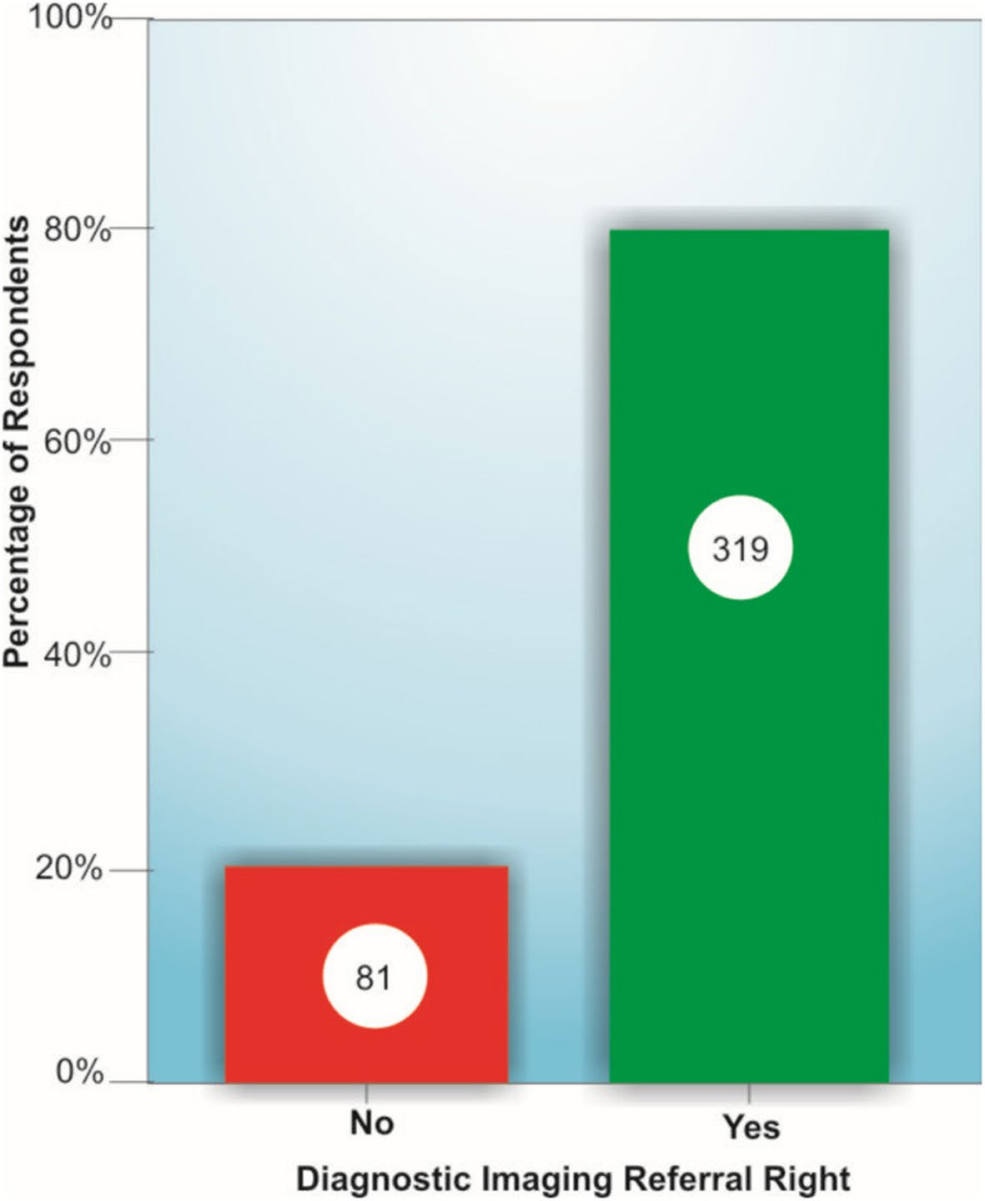
Respondents with MI authority in their practice settings

Of the 2308 eligible participants emailed, 2125 survey invites were successfully delivered, and 436 responses were received (20.5% response rate). We received 54 (2.5%), 103 (4.9%), 239 (11.2%), and 40 (1.9%) responses in the first, second, third, and fourth weeks, respectively. Thirty-six entries with incomplete data were discarded, leaving 400 valid entries, which was adequate for our sample size. Of the 400 participants, the majority (79.8%) reported having a MI authority in their practice settings (Fig. 2). Table 1 showed the participants’ demographics: 68.7% were males, 70.0% had an entry-level bachelor’s degree only, and the majority (81.0%) were within their first decade of practice.

**Table 1:** Participants’ demographic variables

Parameter	***n*** (%) 400 (100%)
**Sex**	
Male	275 (68.7)
Female	125 (31.3)
**Years in practice**	
2–10	324 (81.0)
11–20	49 (12.3)
21–30	27 (6.7)
**Practice setting**	
Federal Hospital	124 (31.0)
State Hospital	74 (18.5)
Private PT Clinic	64 (16.0)
Private Hospital	60 (15.0)
Home Care PT	33 (8.3)
University	19 (4.7)
Sports Team	6 (1.5)
Others	20 (5.0)
**Speciality**	
Musculoskeletal	185 (46.3)
Neurology	86 (21.5)
Cardiopulmonary	20 (5.0)
Paediatrics	27 (6.8)
Sports	26 (6.5)
Women-Health	17 (4.2)
Community PT	15 (3.7)
Geriatrics	8 (2.0)
Others	16 (4.0)
**Highest qualification**	
Bachelor	280 (70.0)
t-DPT	8 (2.0)
Masters	89 (22.3)
Doctorate (Ph.D.)	23 (5.7)

*PT* Physiotherapy, *t-DPT* Transitional doctor of physiotherapy, *Ph.D.* Doctor of Philosophy

### Participants’ levels of training, attitude, utilisation, and competence in musculoskeletal imaging

The overall level of MI training among participants was fair (*Mdn* = 10, *R* = 6–30). There was a significant difference in reported levels of training across MI procedures [Friedman’s ANOVA, χ^2^ (15) = 1285.899, *p* = 0.001]. The post hoc test showed that training in X-ray was better than MRI and CT scan, which were better than ultrasound, bone scan, and DEXA (*p* < 0.05). Specifically, most of the participants (75.7%) had a good, very good, or excellent level of training in interpreting X-rays. Conversely, 69.3, 78.0, 92.8, 94.8, and 95.0% had a fair or poor level of training in the interpretation of MRI, CT scan, ultrasound scan, bone scan, and DEXA, respectively. The details were presented in Table 2.

**Table 2:** Response percentage and median of the levels of MI training (*n* = 400)

Diagnostic imaging modality	Poor (1)	Fair (2)	Good (3)	V. Good (4)	Excellent (5)	Median (1–5)
Radiography (X-ray).	3.5%	20.8%	43.0%	26.2%	6.5%	**3**
Magnetic Resonance Imaging (MRI).	29.3%	40.0%	24.7%	4.7%	1.3%	**2**
Computed tomography (CT scan).	39.0%	39.0%	17.8%	3.5%	0.7%	**2**
Musculoskeletal Ultrasound Scan.	77.0%	15.8%	5.5%	1.5%	0.2%	**1**
Scintigraphy (bone scan).	78.3%	16.5%	3.7%	1.3%	0.2%	**1**
Dual Energy X-Ray Absorptiometry (DEXA or DXA).	84.5%	10.5%	4.3%	0.7%	0.0%	**1**

These are responses to the question “On a scale from 1 to 5, with 1 = poor, 2 = fair, 3 = good, 4 = very good and 5 = excellent, how do you rate your current level of training in referral and utilisation of the following modalities?” Part C: The median (range) of participants’ total score was fair, 10 (6 to 26), expected range = 6 to 30

Table 3 shows that the participants had a positive attitude towards the use of MI in clinical practice (*Mdn* = 32, *R* = 8–40). Most of the participants strongly agreed or agreed that physiotherapists can recognise a need for MI (93.7%), incorporate MI results into critical reasoning for patient care (93.7%), and weigh the benefits and hazards associated with each procedure (76.2%).

**Table 3:** Response percentage and median showing the attitude of the participants towards physiotherapists’ referral and utilisation of MI (*n* = 400)

Item	S.D. (1)	D (2)	I (3)	A (4)	S.A. (5)	Median (1–5)
Physiotherapists are capable of recognising the need for diagnostic imaging in patients.	1.5%	1.5%	3.3%	44.5%	49.2%	**4**
Physiotherapists are capable of incorporating imaging results into initial and subsequent clinical reasoning.	1.8%	2.0%	2.5%	49.3%	44.4%	**4**
Physiotherapists can provide a preliminary clinical examination to verify if imaging will be necessary to arrive at a diagnosis.	2.0%	1.5%	3.3%	45.0%	48.2%	**4**
Physiotherapists are capable of considering cost-effectiveness while referring a patient for diagnostic imaging.	2.5%	5.5%	7.8%	50.5%	33.7%	**4**
Physiotherapists are capable of weighing the benefit of diagnostic imaging modalities against potential hazards from ionising radiation.	2.0%	4.8%	17.0%	47.7%	28.5	**4**
Physiotherapists have the potential to operate real-time musculoskeletal ultrasound imaging to supplement their clinical examination.	6.0%	16.0%	25.5%	32.0%	20.5%	**4**
Physiotherapists are capable of reading and interpreting imaging results accurately.	2.5%	8.5%	15.0%	46.0%	28.0%	**4**
Physiotherapists are not restricted by any Nigerian law or health sector regulation from referring patients for diagnostic imaging.	13.0%	18.5%	19.8%	30.2%	18.5%	**3**

These are responses to the question “On a scale from 1 to 5, with 1 = strongly disagree (S.D.), 2 = disagree (D), 3 = indifference (I), 4 = agree (A) and 5 = strongly agree (S.A.), what is your opinion on the following (statements) items?” Part D: The median (range) of participants’ total score showed a positive attitude, 32 (8 to 40), expected range = 8 to 40

Eighty-eight per cent of participants reported that they use the Clinical Decision Criteria (CDC) to verify if a patient would need diagnostic imaging before referring them to the radiologist. Most participants reported that they never or rarely perform a musculoskeletal ultrasound scan (97.3%) or ordered DEXA (81%) during clinical practice. Table 4 shows that the participants occasionally utilised MI during musculoskeletal assessments (*Mdn* = 21, *R* = 8–40).

**Table 4:** Response percentage and median on utilisation of MI results (*n* = 400)

Item	Never (1)	Rarely (2)	Some times (3)	Most time (4)	Always (5)	Median
You use diagnostic imaging tests for musculoskeletal assessment.	3.8%	3.2%	35.3%	42.5	15.2	**4**
You perform a musculoskeletal ultrasound scan by yourself during patient assessment.	88.5%	8.8%	1.5%	1.0%	0.2%	**1**
You initiate some treatment while awaiting diagnostic imaging result	1.8%	5.5%	50.2%	35.3%	7.2%	**3**
You do not depend on the reports given by the radiologist only *(you review the films).*	2.3%	5.7%	20.8%	31.7%	39.5%	**4**
The outcome of imaging does not change the conservative line of management already adopted for the patient.	8.3%	16.0%	57.0%	17.0%	1.7%	**3**
You send for Dual Energy X-ray Absorptiometry before spinal manipulation in geriatrics.	59.0%	21.8%	9.2%	4.0%	6.0%	**1**
You utilise Clinical Decision Criteria to verify if a patient would need diagnostic imaging before referrals.	3.8%	8.5%	20.2%	40.0%	27.5%	**4**

These are responses to the question “On a scale from 1 to 5, with 1 = never, 2 = rarely, 3 = sometimes, 4 = most time and 5 = always, how do the following statements regarding utilisation of diagnostic imaging, apply to you?” Part E: The median (range) of participants’ total score was 21 (11 to 30), expected range = 7 to 35

Overall, participants were moderately competent in the use of MI (*Mdn* = 16, *R* = 6–30). However, levels of competencies differ across MI procedures [Friedman’s ANOVA, χ^2^ (15) = 1310.769, *p* < 0.001]. The post hoc test showed their competence in X-ray was higher than MRI and CT scans, which were higher than ultrasound scan, bone scan and DEXA, in that order (*p* < 0.05). Table 5 shows most of the participants (81.7%) were competent or very competent in the use of X-rays, whereas 71.5, 72.5, and 77%, reported being incompetent or very incompetent in the use of ultrasound scans, bone scans, and DEXA, respectively. An appreciable number of the participants, 39.0 and 37.3% were moderately competent in the use of CT and MRI scans, respectively.

**Table 5:** Response percentage and median on the levels of competence in MI referral and utilisation (*n* = 400)

Diagnostic imaging modality	V.I. 1	I 2	N 3	C 4	V.C. 5	Median
Radiography (X-ray).	1.5%	2.0%	14.8%	59.2%	22.5%	**4**
Magnetic Resonance Imaging (MRI).	6.0%	19.5%	37.3%	30.0%	7.2%	**3**
Computed tomography (CT scan).	7.0%	26.8%	39.0%	23.0%	4.2%	**3**
Musculoskeletal Ultrasound Scan.	31.8%	39.7%	23.0%	4.0%	1.5%	**2**
Scintigraphy (bone scan).	35.5%	37.0%	19.0%	7.3%	1.2%	**2**
Dual Energy X-Ray Absorptiometry (DEXA or DXA).	37.8%	39.2%	17.3%	4.5%	1.2%	**2**

These are responses to the question “On a scale from 1 to 5, with 1 = very incompetent (V.I.), 2 = incompetent (I), 3 = neutral (N), 4 = competent (C) and 5 = very competent (V.C.), how would you rate your current level of competence in referral and utilising results from the following modalities?” Part F: The median (range) of participants’ total score showed they were moderately competent, 16 (6 to 30), expected range = 6 to 30

### Correlations between training, attitude, competence, utilisation, and years in practice

Table 6 shows Spearman’s correlation (rho) coefficient among levels of training, attitude, competence, utilisation of MI, and years of clinical practice. There was a moderate positive correlation between training and competence (rho = 0.61). Other pairs of the variables had a low positive correlation, except for year-in-practice versus attitude (rho = − 0.07), and utilisation (rho = − 0.02) which had negative coefficients.

**Table 6:** Spearman’s Correlation: between levels of training, attitude, competence and utilisation of MI, and years in practice (*n* = 400)

Parameters	Attitude *Rho* *p*-value	Competence *Rho* *p*-value	Utilisation *Rho* *p*-value	Years in practice *Rho* *p*-value
**Training**	0.19 < 0.001*	0.61 < 0.001*	0.33 < 0.001*	0.18 < 0.001*
**Attitude**		0.21 < 0.001*	0.22 < 0.001*	− 0.07 0.486
**Competence**			0.23 < 0.001*	0.34 < 0.001*
**Utilisation**				−0.02 0.689

* = Spearman’s Correlation Coefficient (rho) was significance (2-tailed) at *p* < 0.05

### Associations and differences in levels of training, attitude, utilisation, and competence in musculoskeletal imaging across the demographic variables

Mann-Whitney *U* test (Table 7) showed a significant sex difference in the levels of training (*Z* = − 2.20, *p* = 0.028) and competence of MI (*Z* = − 2.37, *p* = 0.018); for each parameter, men had higher median than women. Furthermore, the Kruskal Wallis test showed a significant difference in the reported levels of training, competence, and utilisation across the categories of years in practice. The Dunn-Bonferroni post hoc test indicated that the participants who had practised for two decades and more, reported to have acquired more training (*Z* = − 2.52, *p* = 0.036) and competence (*Z* = − 3.35, *p* = 0.002) than those who had practised for a decade or less. Participants that have practised for 10 years or less (*Z* = 4.00, *p* = 0.001) and those that have practised between 21 to 30 years (*Z* = − 4.46, *p* = 0.002) utilised MI more than their counterparts who had 11 to 20 years of practice experience.

**Table 7:** Differences in levels of training, attitude, competence, and utilisation of MI across categories of the demographic variables (*n* = 400)

Parameter	***N***	Training *Median* *Range (6–30)*	Attitude *Median* *Range* (*8–40*)	Utilisation *Median* *Range* (*7–35*)	Competence *Median* *Range* (*6–30*)
**Sex**					
Male	275	11.0	32.0	21.0	16.0
Female	125	10.0	32.0	21.0	14.0
*Z-statistic* (*p*-value)		−2.20 (0.028)*	**−0.03 (0.973)**	**− 0.05 (0.958)**	− 2.37 (0.018)*
**Years in practice**					
2–10	324	10.0	32.0	21.0	15.0
11–20	49	11.0	31.0	19.0	16.0
21–30	27	11.0	33.0	22.0	18.0
*H-statistic* (*p*-value)		8.50 (0.014)*	**3.05 (0.218)**	18.18 (0.001)*	11.43 (0.003)*
**Practice setting**					
Federal Hospital	124	11.0	32.0	20.5	15.0
State Hospital	74	10.0	31.5	21.0	16.0
Private PT Clinic	64	10.0	32.5	21.0	16.0
Private Hospital	60	10.0	32.0	21.0	16.0
Home Care PT	33	10.0	32.0	21.0	16.0
University	19	12.0	29.0	19.0	15.0
Sports Team	6	10.0	31.0	19.0	18.0
Others	20	10.0	32.5	20.5	14.0
*H-statistic* (*p*-value)		**7.32 (0.396)**	**3.24 (0.862)**	**10.45 (0.164)**	**10.23 (0.176)**
**Speciality**					
Musculoskeletal	185	10.0	29.5	20.0	15.0
Neurology	86	11.0	32.0	21.5	15.0
Cardiopulmonary	20	11.0	31.0	20.0	16.0
Paediatrics	27	12.0	32.0	21.0	16.0
Sports	26	9.0	32.0	21.0	15.0
Women-Health	17	8.0	32.0	21.0	14.0
Community PT	15	11.0	29.0	18.0	13.0
Geriatrics	8	11.0	34.0	20.0	16.0
Others	16	10.0	34.5	20.0	15.0
*H-statistic* (*p*-value)		**10.14 (0.256)**	**13.88 (0.085)**	16.03 (0.042)*	16.48 (0.036)*
**Highest qualification**					
Bachelor	280	10.0	32.0	21.0	15.0
t-DPT	8	18.5	32.0	21.0	21.5
Masters	89	10.0	31.0	19.0	16.0
Doctorate (Ph.D.)	23	11.0	32.0	20.0	18.0
*H-statistic* (*p*-value)		12.87 (0.005)*	**6.18 (0.103)**	9.76 (0.021)*	11.25 (0.010)*
**MI authority**					
Yes	319	10.0	32.0	21.0	15.0
No	81	10.0	30.0	20.0	16.0
*Z-statistic* (*p*-value)		**−0.98 (0.325)**	−2.93 (0.003)*	−2.47 (0.013)*	**−0.52 (0.605)**

^*^ = Z or H-statistic was significant at *p* < 0.05 level (2-tailed). Mann-Whitney U test was reported as Z-statistics. PT = Physiotherapy. t-DPT = transitional doctor of physiotherapy. Ph.D. = Doctor of Philosophy. MI = musculoskeletal imaging. Training scores ≤6 = poor, 7 to 12 = fair, 13 to 18 = good, 19 to 24 = very good, and 25 to 30 = excellent training. Attitude scores 8 to 16 = negative, 17 to 24 = neutral, and 25 to 40 = positive attitude. Competence scores ≤6 = very incompetent, 7 to 12 = incompetent, 13 to 18 = moderately competent, 19 to 24 = competent, and 25 to 30 = very competent. Utilisation scores ≤7 = never, 8 to 14 = rarely, 15 to 21 = sometimes, 22 to 28 = most time, and 29 to 35 = always use musculoskeletal imaging

The practice setting did not have a significant influence on any of the parameters (Table 7). However, there was a significant difference in the levels of competence and utilisation of MI across specialities. The post hoc test showed that participants from the musculoskeletal speciality reported higher levels of competence (*Z* = 3.24, *p* = 0.001) and utilisation of MI (*Z* = 2.56, *p* = 0.01) than their counterparts in the Women-health speciality, and there was no significant difference between other specialities.

Higher academic qualifications had a significant influence on the reported levels of training, competence, and utilisation of MI. The post hoc test showed that participants with a bachelor of physiotherapy degree, reported a lower level of MI training (*Z* = − 2.66, *p* = 0.047), competence (*Z* = − 2.62, *p* = 0.05), and utilisation (*Z* = − 2.72, *p* = 0.04) than their counterparts with transitional Doctor of Physiotherapy (t-DPT) degree. Participants who had MI authority in their practice settings reported higher levels of positive attitude (*Z* = − 2.93, *p* = 0.003), and utilisation of MI (*Z* = − 2.47, *p* = 0.013), than those without referral-right (Table 7).

## Discussion

The study explored the MI authority, levels of training, attitude, competence, and utilisation among physiotherapists in Nigeria. Participants had a positive attitude towards MI, their level of training was fair, while their competence and use of MI were moderate. Specifically, this study found that participants’ level of training in X-ray was good, while MRI and CT scans were fair, training in ultrasound scan, bone scan, and DEXA were poor. The finding was consistent with a study conducted in a USA-based institution, there was a considerable bias towards instructions in X-ray and MRI when compared to CT and ultrasound scans [25]. Deficiencies in physiotherapists’ MI education may have a negative implication for quality of care [16].

Corresponding with the levels of training on MI procedures, many participants reported that they were incompetent in the application of scintigraphy, DEXA and real-time musculoskeletal ultrasound scan which are now commonly used in physiotherapy practice [3, 26]. Similarly, a Canadian survey reported that about three-quarters of post-licensure physiotherapists in Ontario were incompetent with MRI, CT, and ultrasound scans [15]. Furthermore, most UK respondents rated themselves as “not at all competent” in musculoskeletal sonography [27]. Following the evidence of MI competence among USA military physiotherapists with advanced training, in 2014, Boissonnault et al. [14] recommended an upgrade of the entry-level MI physiotherapy curriculum to prepare practitioners for MI authority.

The participants in the current study had a positive attitude towards MI. Similarly, Little and Lazaro [28] reported that a cohort of USA-based physiotherapists had a positive perception of the utilisation of diagnostic imaging reports (DIR). However, the present study found a negative correlation between participants’ attitudes towards MI and years in practice. Some older physiotherapists believed that MI is beyond the current scope of physiotherapy practice in Nigeria, others thought that access to DIR is enough. The benefit of MI in physiotherapy goes beyond diagnosis, it is also useful for follow-up [8]. For instance, ultrasonography can be used to monitor real-time changes in morphology and biomechanics of muscles, follow up progression in muscle and joint pathologies, and conduct ultrasound-guided procedures [3, 12, 26, 29].

Most participants in our study reported that they used imaging for musculoskeletal assessment. Little and Lazaro [28] reported that if available, 83.4% of their participants utilised DIR. The reader should note the difference between having the right to order MI and having access to DIR already processed through a physician’s directive [9]. The benefits of granting physiotherapists MI referral-right include enhancing their autonomy, better clinical decision-making, and reducing patients’ waiting time [3, 8].

Also, the current study found some notable MI practices among the participants such as screening the patients with appropriate CDC before MI referral. The use of the CDC, including the American College of Radiology Appropriateness Criteria, are foundations for deciding on the appropriateness of ordering an MI procedure [30–32]. Moreover, some participants often reviewed the printed images before applying the accompanying report. Although radiologists are responsible for reporting diagnostic imaging, it is recommended that physiotherapists juxtapose the films, DIR, medical history, and physical examination for comprehensive assessment [30]. The current authors align with scholars who posit that MI should not be misconstrued as an alternative to detailed medical history and clinical examination [3, 8, 30, 31]; rather, it should be used as an adjunct for broadening clinical assessment and decision-making.

Approximately 80% of the participants reported that they have musculoskeletal imaging referral rights. Mabry et al. [6] examined physiotherapists’ MI authority in 81 countries (WCPT member nations); only one-third of the countries had affirmative policies. Among the countries were African nations such as Ethiopia and Zambia with full MI authority. Others were Benin, Ghana, Swaziland, Malawi, South Africa, Rwanda, Tanzania, and Zimbabwe with partial authority covering mostly, the use of plain X-ray and MRI [6]. Although the majority of the participants reported having MI authority in their practice settings, the MRTB (Physiotherapy Practice) Act 2004 neither affirmed nor restricted Nigerian physiotherapists from MI referral [13]. Notwithstanding that the MRTB Act [13] and the Nigerian Radiation Safety in Diagnostic and Interventional Radiology Regulations [33], did not include any prohibitive language against physiotherapists ordering MI of patients, it is important to pass formal legislation on physiotherapist MI authority in Nigeria.

The implication of this study for physiotherapy education is that despite the evidence of MI competence among advanced practitioners [8], our study and others [14, 25]  found a need to improve the entry-level training in musculoskeletal ultrasonography, scintigraphy, and DEXA. The WCPT should encourage each member nation to conduct a comprehensive review of MI educational needs, competencies, rights, and privileges to ensure the quality of care and as a benchmark for future curriculum upgrade and practice scope legitimisation advocacy.

### Limitations

The voluntary response sampling technique and low response rate were factors that could affect the generalisability of our findings due to the potential for nonresponse bias [23]. However, these factors may be of less impact, since the desired sample size and data collection duration were met [21]. Nonetheless, our sample size and demographic distribution were similar to previous studies on physiotherapists in Nigeria [2, 18, 19]. Furthermore, the study instrument was designed to generate subjective data based on self-reports, using a simple five-point Likert scale. Subjectivity is a general limitation of questionnaire-based studies [12]. In this case, there could be potential for social desirability bias when physiotherapists were asked to rate their own attitudes or competencies.

## Conclusion

The participants had a positive attitude towards the use of MI results to aid clinical decision-making. The majority of the participants assumed that they have MI referral-right in their practice settings, but the Practice Act was silent on physiotherapists’ MI authority. Explicitly, the participants had access to MI reports in patients’ records, but they may rely on physicians’ directives for MI referrals. The level of training, competence, and utilisation of plain radiography, magnetic resonance imaging, and computed tomography procedures were significantly higher than bone scan, dual-energy X-ray absorptiometry, and procedural ultrasound. Therefore, curriculum contents for scintigraphy, dual-energy X-ray absorptiometry, and ultrasonography should be upgraded. Physiotherapists’ MI authority in Nigeria should be formalised through legislation, to improve efficiency in service delivery and quality of care.

## Data Availability

The questionnaire used for this study is available at 10.4102/sajp.v75i1.1338. The dataset generated and analysed during the current study will be made public by 2023 but is available from the corresponding author on a reasonable request. This is in adherence to our institutional policy of holding data for 5 years after study completion.

## Abbreviations

CDC: Clinical Decision Criteria
CT scan: Computed Tomography Scan
DEXA: Dual Energy X-ray Absorptiometry
DIR: Diagnostic Imaging Report
DPT: Doctor of Physical Therapy
MI: Musculoskeletal Imaging
MRI: Magnetic Resonance Imaging
MRTB: Medical Rehabilitation Therapists Board of Nigeria
PMIPQ: Physiotherapist’s Musculoskeletal Imaging Profiling Questionnaire
t-DPT: Transitional Doctor of Physical Therapy Programme
UK: United Kingdom
USA: United States of America
WCPT: World Physiotherapy (formerly: World Confederation for Physical Therapists)
X-ray: Radiography
